# Prevalence of CTX-M types among ESBL-producing pathogenic *Escherichia coli* isolates from foodborne diarrheal patients in Gyeonggi-do, South Korea

**DOI:** 10.1007/s10068-024-01549-5

**Published:** 2024-04-24

**Authors:** Nanjoo Park, Jeong In Hur, Sohyun Lee, Sangryeol Ryu

**Affiliations:** 1 Department of Food and Animal Biotechnology, Seoul National University, Seoul, South Korea; 2 Department of Agricultural Biotechnology, Seoul National University, Seoul, South Korea; 3 Research Institute of Agriculture and Life Sciences, Seoul National University, Seoul, South Korea; 4 Center for Food and Bioconvergence, Seoul National University, Seoul, South Korea; 5 Gyeonggi-do Research Institute of Health & Environment, Suwon, South Korea

**Keywords:** Pathogenic *Escherichia coli*, Antibiotic resistance, Extended-spectrum β-lactamase (ESBL), Plasmid-mediated quinolone resistance genes (PMQR), Foodborne outbreaks

## Abstract

Prevalence and characteristics of extended-spectrum β-lactamase (ESBL)-producing pathogenic *Escherichia coli* from foodborne diarrheal patients were studied. Analysis of 495 *E. coli* isolates revealed that 80 isolates were ESBL-producing pathogenic *E. coli*, and enteroaggregative *E. coli* and enterotoxigenic *E. coli* were two of the most prevalent pathotypes. In silico Clermont phylo-typing of the 80 ESBL-producing *E. coli* showed that phylogroup A (49/80) and D (22/80) were the predominant phylogroups. The average nucleotide identity analysis of ESBL-producing *E. coli* disclosed that they could be grouped into two phylogenetic groups; 25 A and 55 B groups. All strains, except one, harbored the *bla*CTX-M gene. All CTX-M-15 type ESBL-producing strains also carried *qnrS*, a plasmid-mediated quinolone resistance gene (PMQR). These results suggest that the diversity of ESBL-producing *E. coli* is high and that co-existence of *bla*CTX-M-15 and *qnrS* genes is widespread, highlighting their high risk of antibiotic-resistance spreading in infectious disease outbreaks.

**Supplementary Information:**

The online version contains supplementary material available at 10.1007/s10068-024-01549-5.

## Introduction

Even though many countries have food safety guidelines and foodborne disease regulations, large-scale foodborne disease outbreaks continue to increase worldwide (Lee & Yoon, 2021). Foodborne diseases can be divided into two categories, infection or poisoning, which can be further categorized according to etiological agents such as viruses, bacteria, parasites, and chemicals (Bari & Yeasmin, 2018). Among these agents, pathogenic *Escherichia coli* is one of the most common cause of foodborne disease outbreaks reported in the United States, Japan, and South Korea (Dewey-Mattia et al., 2018; Lee & Yoon, 2021). The Ministry of Food and Drug Safety of South Korea estimates that 5,582 outbreaks of foodborne-related illness occurred in South Korea from 2002 to 2022, resulting in 139,630 patients suffering from foodborne diseases. Norovirus (29.4%) and pathogenic *E. coli* (22.2%) are two of the most frequently reported etiological agents associated with foodborne disease outbreaks in South Korea (https://www.foodsafetykorea.go.kr/). The most serious outbreak of *E. coli*-caused foodborne illness was reported in Germany in 2011, which subsequently spread to many other countries, resulting in 3,816 identified cases of enterohemorrhagic *E. coli* infection and 54 associated deaths worldwide (Buchholz et al., 2011; Frank et al., 2011).

Pathogenic *E. coli* are categorized into the five pathotypes based on virulence factors, toxin production, host cell attachment, and invasiveness; enteropathogenic *E. coli* (EPEC), enterohemorrhagic *E. coli* (EHEC), enteroinvasive *E. coli* (EIEC), enterotoxigenic *E. coli* (ETEC), and enteroaggregative *E. coli* (EAEC) (Yang et al., 2017). Previous studies in South Korea, Qatar, and Iran have reported that most pathogenic *E. coli* strains from foodborne disease outbreaks are resistant to third-generation cephalosporins (Aminshahidi et al., 2017; Eltai et al., 2020; Kim et al., 2014). Although antibiotics remain the most commonly used treatment against pathogenic *E. coli* in clinical cases, excessive use of these antimicrobials has increased the emergence of multidrug-resistant strains (Pasberg-Gauhl, 2014). According to the Centers for Disease Control and Prevention, over 2.8 million individuals become infected with antibiotic-resistant pathogens each year in the United States, and over 35,000 die as a consequence of these infections (CDC, 2019). The emergence and rapid spread of *E. coli* carrying genes encoding extended-spectrum β-lactamases (ESBLs) or carbapenemases are considered urgent health problems worldwide (Zhang et al., 2018). ESBLs are a group of plasmid-encoded enzymes that hydrolyze β-lactams, including penicillin, cephalosporins, and monobactam, but not carbapenem (Park et al. 2019; Rawat & Nair 2010). Derivatives of ESBL genes including *bla*TEM (named after patient Temoniera), *bla*SHV (sulfhydryl variable active site), *bla*CTX (cefotaximase)-M, and *bla*CMY (cephalomycinase) are known and their distribution is geographically different (Pitout & Laupland, 2008; Hawkey & Jones, 2009; Lalak et al., 2016; Ghenea et al., 2022). CTX-M-type ESBLs have spread rapidly among *E. coli* strains, with the *bla*CTX-M-15 gene being identified as the predominant type (Carvalho et al., 2021).

In this study, to investigate the distribution of ESBLs and their genomic diversity in ESBL-producing pathogenic *E. coli* strains isolated from patients with food poisoning, we examined the resistance of 80 ESBL-producing pathogenic *E. coli* strains to various antibiotics and analyzed their genomic diversities by whole genome sequencing (WGS). This information will help us to better understand the molecular epidemiology of antimicrobial-resistant pathogenic *E. coli* isolated from foodborne disease outbreaks.

## Materials and methods

### Isolation and identification of ESBL-producing pathogenic *E. coli*

A total of 495 pathogenic *E. coli* strains, including 80 ESBL-producing pathogenic *E. coli*, were isolated from the diarrhea patients (one sample per person) by standard rectal swab sample collection method in Gyeonggi-do, South Korea, by the Research Institute of Health & Environment from 2014 to 2018. General information on 80 ESBL-producing pathogenic *E. coli* strains was listed in Table S1. For isolation of pathogenic *E. coli*, each swab sample was incubated at 37 °C for 16 h in tryptone soy broth (TSB; Oxoid, Basingstoke, UK). An aliquot of each TSB enrichment was streaked on MacConkey Agar (Oxoid, Basingstoke,UK) and incubated at 37 °C for 24 h. The pink single colonies were sub-cultures on tryptic soy agar (TSA; Oxoid, Basingstoke,UK) and confirmed using the VITEK 2 system with gram-negative (GN) identification card (bioMérieux, Marcy, France). For screening of ESBL-producing pathogenic *E. coli* strains, identified *E. coli* strains were cultured on CHROMagar ESBL (CHROMagar, Paris, France) at 37 °C for 24 h. The dark pink to reddish single colonies were sub-cultured on TSA (Oxoid). ESBL production was confirmed using VITEK 2 system with AST-N169 card (bioMérieux).

### Pathotype determination of pathogenic *E. coli*

The presence of virulence genes associated with pathogenic properties of *E. coli* were determined by PCR amplification. The isolates were cultured in TSB at 37 °C for 24 h, and their genomic DNA was extracted from overnight cultures of pathogenic *E. coli* isolates using the Nextractor NX-48 system and NX-48 bacterial DNA kits (Genolution Inc., Seoul, Korea). PCR was performed using PowerChek™ Diarrheal *E. coli* 8-plex Detection Kit (Kogenbiotech, Seoul, Korea) according to the manufacturer’s instructions. The amplified virulence genes of pathogenic *E. coli* strains determined ETEC (*st* and *lt* encoding heat-stable and heat-labile enterotoxins), EHEC (VT1 and VT2 encoding verocytotoxin 1 and 2), EPEC (*eaeA* encoding intimin), EAEC (*aggR* encoding transcription regulator for aggregative adherence fimbria I), and EIEC (*ipaH* encoding invasion plasmid antigen H).

### Antimicrobial susceptibility tests

The antimicrobial susceptibility tests were performed using the VITEK 2 system with AST-N169 card (bioMérieux) according to the manufacturer’s instructions. *Escherichia coli* ATCC 25922 was used as a control strain. The AST-N169 card were used for 17 antibiotics tests; ampicillin (AMP), amoxicillin/clavulanic acid (AMC), ampicillin/sulbactam (SAM), cefalotin (CEF), cefazolin (CFZ), cefotetan (CTT), cefoxitin (FOX), cefotaxime (CTX), ceftriaxone (AXO), imipenem (IMI), amikacin (AMK), gentamicin (GEN), nalidixic acid (NAL), ciprofloxacin (CIP), tetracycline (TET), chloramphenicol (CHL), trimethoprim/sulfamethocxazole (SXT). The interpretation of resistant (R), intermediate (I) and sensitive (S) was based on the criteria issued by the Clinical and Laboratory Standards Institute (CLSI, 2021). The minimum inhibitory concentrations (MIC) of ciprofloxacin, tetracycline, chloramphenicol and trimethoprim/sulfamethocxazole were determined using the E-test method (bioMerieux Inc.). Fluoroquinolone, tetracyclines, phenicol, and folate pathway inhibitor MIC values were confirmed according to Clinical and Laboratory Standards Institute guidelines (CLSI, 2021) and European Committee on Antimicrobial Susceptibility Testing guidelines (EUCAST, 2022).

### Whole genome sequencing

All ESBL-producing pathogenic *E. coli* strains (n = 80) were subjected to whole genome sequencing. Genomic DNA of the isolates was extracted a NucleoSpin Microbial DNA kit (Macherey–Nagel, USA) and TissueLyser II (Qiagen, Germany) by following the manufacturer’s instructions. The quality of genomic DNA was determined by NanoDrop spectrophotometer (Thermo-Fisher Scientific, USA), standard agarose gel electrophoresis, and the Qubit 3.0 fluorometer (Thermo-Fisher Scientific). Intact genomic DNA was sheared by a Covaris S220 ultra sonicator (Covaris, USA), and the sequencing library was constructed using the Illumina TruSeq Nano DNA LT library prep kit (Illumina, USA) according to the TruSeq Nano DNA Library Preparation protocol. The quality of the libraries was assessed on a 2100 Bioanalyzer System with DNA 1000 Chip (Agilent Technologies, USA). Sequencing was performed using the NextSeq 500 Sequencing System (Illumina, USA) to generate 2 × 150 bp read length. The contigs of genomic sequences were de novo assembled using CLC Genomics Workbench v20 (Qiagen, USA) with default parameters.

### Bioinformatics analysis

To analyze draft genome of each strain, annotation of the assembled genomes was performed using Prokka Web-based tool v1.12 (https://github.com/tseemann/prokka). STs were determined using MLST 2.0 (https://cge.food.dtu.dk/services/MLST/) with 7 housekeeping *E. coli* genes (*adk, fumC, gyrB, icd, mdh, purA,* and *recA*) (Larsen et al., 2012). Antibiotics Resistance Genes (ARGs) were identified using the ResFinder 4.1 database (https://cge.food.dtu.dk/services/ResFinder/) (Bortolaia et al., 2020). Phylogroup was assigned with ClermonTyping (http://clermontyping.iame-research.center/). The *E. coli* strains were divided into seven main phylogroups termed A, B1, B2, C, D, E and F (Beghain et al., 2018). To specify the degree of overall relatedness among genomes, we estimated the genome-wide ANI using FastANI v1.33. ANI analysis estimates the average nucleotide identity of all orthologous genes shared between any two genomes. Organisms belonging to the same species typically exhibit ≥ 95% ANI (Goris et al., 2007; Jain et al., 2018). Pairwise ANI values were visualized using a heat map generated by ComplexHeatmap v2.2.0 and gplots v3.3.5 in R, dividing the strains into four phylogenetic clusters.

## Results and discussion

### Isolation of ESBL-producing pathogenic *E. coli*

A total of 495 pathogenic *E. coli* strains were isolated from 1,901 clinical specimens obtained from diarrhea patients of foodborne outbreaks in Gyeonggi-do, South Korea from 2014 to 2018. The presence of virulence genes associated with pathogenesis of *E. coli* was determined by PCR amplification and they could be classified into four pathotypes based on the presence of virulence genes; 254 (51.3%) isolates were ETEC having *st/lt*, 126 (25.5%) isolates were EAEC having *aggR*, 111 (22.4%) isolates were EPEC having *eaeA*, and 4 (0.8%) isolates were EHEC having *vt1/vt2*. However, EIEC having an *ipaH* was not detected (Table 1). ETEC and EAEC were identified as the two most frequently isolated *E. coli* pathotypes, consistent with the previous reports from Iran (Alizade et al., 2019) and Bangladesh (Fahim et al., 2018). The hybrid strains of pathogenic *E. coli*, which contain multiple virulence genes that may confer higher virulence, have recently been reported worldwide (Santos et al., 2020). However, no hybrid strains were detected in the present study. Antibiotics resistance of the 495 pathogenic *E. coli* isolates were tested by CHROMagar ESBL selective medium and 80 isolates (16.2%) showed resistance to β-lactam antibiotics. The pathotypes of these 80 β-lactam-resistant isolates were 44 ETEC, 26 EAEC, and 10 EPEC. But none of them were EHEC or EIEC (Table 1). The prevalence of ESBL-producing pathogenic *E. coli* in this study was similar to the previously published data from South Korea and China (Song et al., 2009; Xu et al., 2018). These results highlight the diverse distribution of pathogenic *E. coli* pathotypes in diarrhea patients in Gyeonggi-do, South Korea. Moreover, the emergence of antibiotic resistance, particularly the 16.2% resistance to ESBLs, emphasizes the need for continuous monitoring and surveillance.

**Table 1:** Distribution of pathotypes among pathogenic *E. coli* and ESBL-producing pathogenic *E. coli* isolated from foodborne disease patients

Year	No. of isolated pathogenic *E. coli* (n = 495)				No. of isolated ESBL-producing pathogenic *E. coli* (n = 80)			
	EAEC	EPEC	ETEC	EHEC	EAEC	EPEC	ETEC	EHEC
2014	19	29	30	0	9	1	0	0
2015	2	21	58	1	0	3	0	0
2016	0	4	46	1	0	0	24	0
2017	73	31	30	1	17	4	19	0
2018	32	26	90	1	0	2	1	0
Total	126	111	254	4	26	10	44	0

*EAEC,* enteroaggregative *E. coli*; *EPEC,* enteropathogenic *E. coli*; *ETEC,* enterotoxigenic *E. coli*; *EHEC,* enterohemorrhagic *E. coli*

### Antimicrobial susceptibility of the ESBL-producing *E. coli*

The antimicrobial susceptibilities of 80 ESBL-producing pathogenic *E. coli* isolates were tested with 17 antibiotics as described in the Materials and Methods and analyzed the results according to CLSI criteria. All tested isolates showed resistance or intermediate resistance to penicillins (e.g., ampicillin), first-generation cephalosporins (e.g., cefalotin and cefazolin), and third-generation cephalosporins (e.g., cefotaxime and ceftriaxone), whereas only four isolates showed resistance or intermediate resistance to second-generation cephalosporins (e.g., cefotetan and cefoxitin). All ESBL-producing pathogenic *E. coli* isolates were susceptible to carbapenems (e.g., imipenem) (Table S2). While bacteria with resistance to three or more categories of antibiotics were defined as multidrug resistance (MDR) (Nath et al., 2020; Magiorakos et al., 2012), all ESBL-producing pathogenic *E. coli* isolates examined in this study showed multidrug resistance to five or more antibiotics. Interestingly, 32 out of 44 of the ETEC strains (72.7%, 32/44) showed resistance to five antibiotics with an AMP-CEF-CFZ-CTX-AXO pattern and 12 EAEC strains (46.2%, 12/26) showed resistance to eight antibiotics with an AMP-SAM-CEF-CFZ-CTX-TET-CHL-SXT pattern (Table 2). Remarkably, all isolates displayed multidrug resistance to five or more antibiotics, and EAEC/ETEC pathotype-specific resistance was observed.

**Table 2:** Multidrug resistance patterns of 80 ESBL-producing pathogenic *E. coli*

No. of antibiotics	Resistance patterns	No. of isolates		
		EAEC (n = 26)	EPEC (n = 10)	ETEC (n = 44)
5	AMP-CEF-CFZ-CTX-AXO	9	2	32
6	AMP-SAM-CEF-CFZ-CTX-TET	0	1	0
6	AMP-CEF-CFZ-AXO-NAL-TET	0	0	1
6	AMP-CEF-CFZ-CTX-AXO-SXT	0	3	0
7	AMP-SAM-CEF-CFZ-CTX-AXO-NAL	1	0	0
7	AMP-SAM-CEF-CFZ-TET-CHL-SXT	1	0	0
7	AMP-CEF-CFZ-FOX-CTX-NAL-CIP	0	0	1
8	AMP-SAM-CEF-CFZ-CTX-TET-CHL-SXT	12	0	6
9	AMP-SAM-CEF-CFZ-CTX-AXO-NAL-TET-SXT	0	1	0
9	AMP-SAM-CEF-CFZ-CTX-AXO-TET-CHL-SXT	2	2	1
10	AMP-AMC-SAM-CEF-CFZ-FOX-NAL-TET-CHL-SXT	0	1	0
10	AMP-SAM-CEF-CFZ-CTX-AXO-GEN-NAL-CHL-SXT	1	0	0
11	AMP-SAM-CEF-CFZ-CTX-AXO-GEN-NAL-CIP-TET-SXT	0	0	2
14	AMP-AMC-SAM-CEF-CFZ-FOX-CTX-AXO-GEN-NAL-CIP-TET-CHL-SXT	0	0	1

AMP, ampicillin; AMC, amoxicillin/clavulanic acid; SAM, ampicillin/Sulbactam; CEF, cefalotin; CFZ, cefazolin; CTT, cefotetan; FOX, cefoxitin; CTX, cefotaxime; AXO , ceftriaxone; IMI, imipenem), AMK, amikacin; GEN, gentamicin; NAL, nalidixic Acid; CIP, ciprofloxacin; TET, tetracycline; CHL, chloramphenicol; SXT, trimethoprim/sulfamethocxazole

### Genetic diversity of ESBL-producing pathogenic *E. coli*

**Fig. 1:**
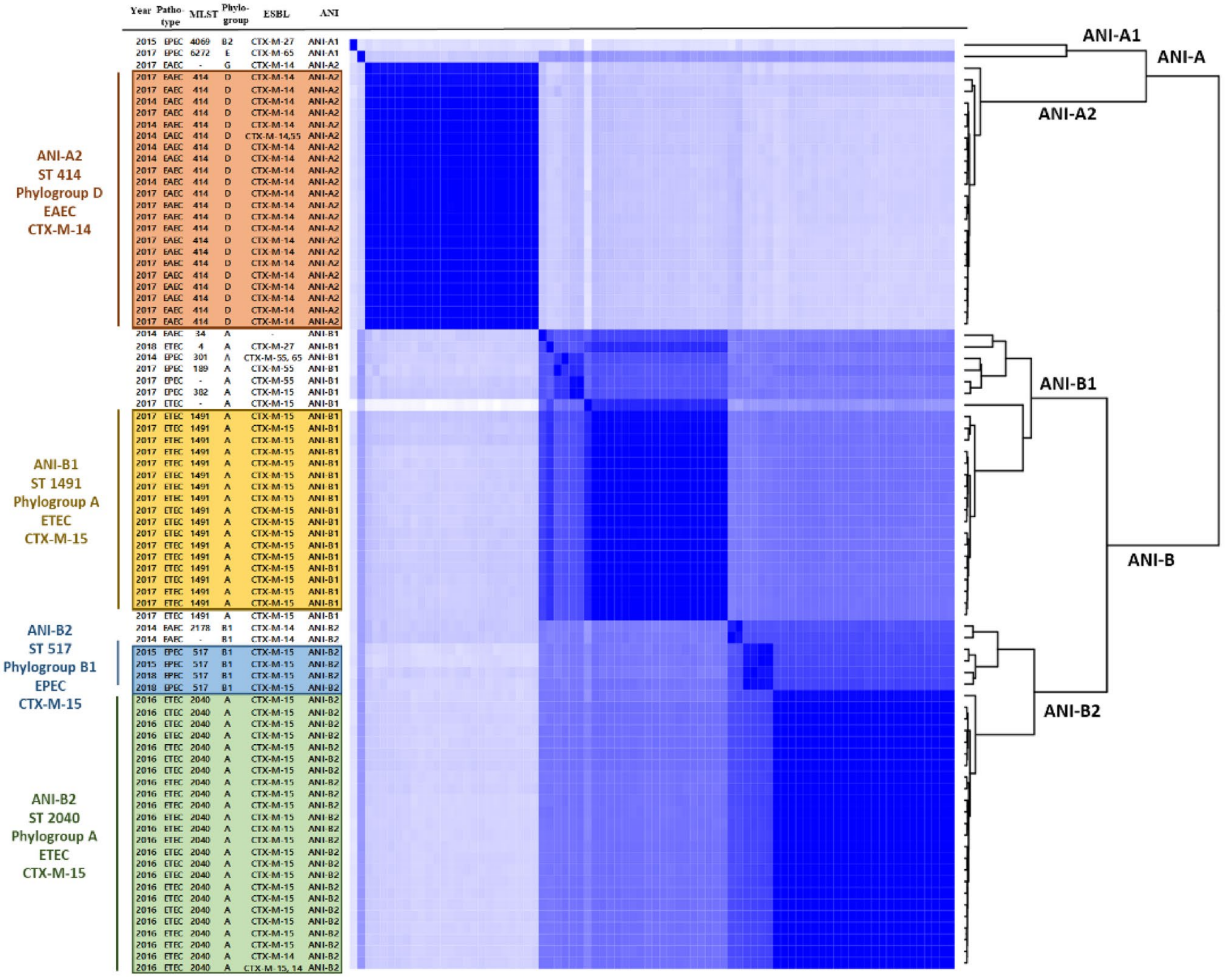
ANI phylogenetic tree of 80 ESBL-producing pathogenic *E. coli* clinical isolates from foodborne disease in Gyeonggi-do. ESBL-producing pathogenic *E. coli* strains are grouped into two major groups (**A** and **B**). The information about pathotypes, MLST, and phylogroups for 80 *E. coli* isolates is indicated on the left side

To analyze genetic diversity of ESBL-producing pathogenic *E. coli*, whole genome sequencing (WGS) and comparative phylogenetic analysis were performed. For this study, multilocus sequencing typing (MLST), phylogroup analysis, and average nucleotide identity (ANI) analysis based on WGS data were conducted. Bioinformatic analysis of the genomes of 80 ESBL-producing pathogenic *E. coli* strains revealed that they have an average genome size of 5,346,277 bp, with 1,450 coding sequences (CDSs), seven rRNA genes, and 83 tRNA genes. MLST analysis of 80 ESBL-producing pathogenic *E. coli* strains using seven house-keeping genes showed that they could be classified into 12 MLST sequence types, including sequence type (ST)4, ST34, ST189, ST301, ST382, ST414, ST517, ST1491, ST2040, ST2178, ST4069, and ST6272. However, four strains could not be classified into any sequence type. Most *E. coli* pathotypes were classified into specific MLST sequence types with exception of 4 strains: most EAEC isolates (n = 26) were ST414 (84.6%, 22/26), most ETEC isolates (n = 44) were either ST2040 (54.5%, 24/44) or ST1491 (40.9%, 18/44), but only 40% (4/10) of EPEC isolates (n = 10) were ST517 (Table 3). The Clermont phylo-typing analysis revealed that phylogroup A (61%, 49/80) and D (28%, 22/80) were the predominant phylogroup in 80 ESBL-producing pathogenic *E. coli* strains. Phylogroup A and D of *E. coli* have been reported to be the most prevalent in patients with diarrhea in India, Sweden, and Spain (Ljungquist et al., 2020; Modgil et al., 2020; Valverde et al., 2009). Phylogroups also showed an association with *E. coli* pathotypes. All ETEC isolates belong to phylogroup A (100%, 44/44), most EAEC isolates belong to phylogroup D (85%, 22/26), and EPEC isolates belong to phylogroups A/B1 (40%, 4/10) respectively (Table 3). The ANI phylogenetic trees of 80 ESBL-producing pathogenic *E. coli* strains showed that two phylogenetic groups were distinctly separated below the 95% ANI threshold: A and B groups (A, 25/80 and B, 55/80). The A group strains were divided into A1 (2 EPEC strains) and A2 (23 EAEC strains), and B group strains were divided into B1 (1 EAEC, 4 EPEC, and 20 ETEC strains) and B2 (2 EAEC, 4 EPEC, and 24 ETEC strains) (Fig. 1). The analysis of genetic diversity in 80 ESBL-producing pathogenic *E. coli* strains revealed a strong phylogenetic association among different *E. coli* pathotypes.

**Table 3:** Distribution of *E. coli* pathotypes in the MLSTs and phylogroups of 80 ESBL-producing pathogenic *E. coli* strains

*E. coli* pathotypes		MLST (No. of strains)	Phylogroups (No. of strains)
EAEC	(n = 26)	ST34 (1), ST414 (22), ST2178 (1), ND (2)	Group A (1), Group B1 (2), Group D (22), Group G (1)
EPEC	(n = 10)	ST189 (1), ST301 (1), ST382 (1), ST517 (4), ST4069 (1), ST6272 (1), ND (1)	Group A (4), Group B1 (4), Group B2 (1), Group E (1)
ETEC	(n = 44)	ST4 (1), ST1491 (18), ST2040 (24), ND (1)	Group A (44)

### Analysis of antimicrobial resistance genes of ESBL-producing pathogenic *E. coli*

**Fig. 2:**
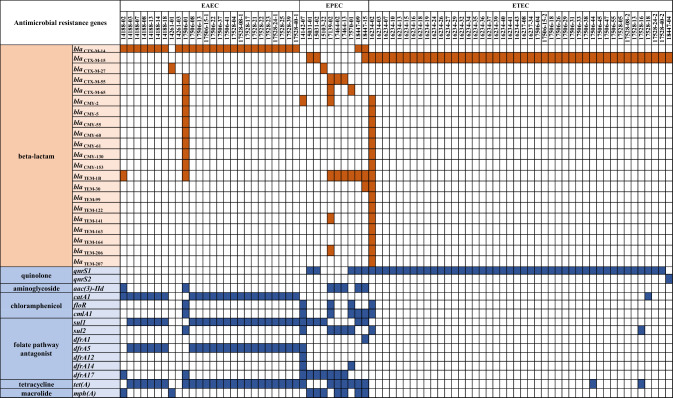
Distribution of antimicrobial resistance (AMR) genes in ESBL-producing pathogenic *E. coli* isolates. The orange color indicates the presence of β-lactamase genes and blue color indicates the presence of other antimicrobial resistance genes based on ResFinder

Analysis of the *bla* genes of 80 ESBL-producing pathogenic *E. coli* isolates revealed following families of β-lactamases: *bla*CTX-M (− 14, 15, 27, 55, and 65), *bla*CMY (− 2, 5, 55, 60, 61, 130 and 153), and *bla*TEM (− 1B, 30, 99, 122, 141, 163, 164, 206, and 207). All these isolates, except one, harbored the *bla*CTX-M gene alone or in combination with either *bla*TEM gene or *bla*CMY gene, suggesting that the *bla*CTX-M gene is the most prevalent among pathogenic *E. coli* from foodborne illnesses (98.8%, 79/80). Prevalence of the *bla*CTX-M variants were as follows; 47 isolates have *bla*CTX-M-15 (44 ETEC and 3 EPEC), 27 isolates have *bla*CTX-M-14 (25 EAEC and 2 EPEC), 4 isolates have *bla*CTX-M-55 (1 EAEC and 3 EPEC), 2 isolates have *bla*CTX-M-27 (1 EAEC and 1 EPEC), and 3 isolates have *bla*CTX-M-65 (1 EAEC and 2 EPEC). In addition, one EPEC strain was found to have both *bla*CTX-M-14 and *bla*CTX-M-15 genes while one EAEC strain have *bla*CTX-M-14, *bla*CTX-M-55 and *bla*CTX-M-65 genes (Fig. 2). It is interesting to note that *E. coli* strains in the ANI-A2 group showed a significantly higher proportion of ST414 and phylogroup D, with *bla*CTX-M-14 being predominant in this group. ST1491 and ST2040 were highly prevalent in the ANI-B group (B1/B2) and in phylogroup A, and *bla*CTX-M-15 was widespread in this group. Phylogenetic studies have demonstrated that ST1491 and ST2040 are closely related. EAEC strains were associated with ANI-A group and ETEC strains were related to the ANI-B group. These results suggest that each *E. coli* pathotype has distinctive phylogenetic background.

The CTX-M-type ESBLs in *E. coli* are the most predominant and rapidly disseminated type in both humans and animals (Smet et al., 2010; Carvalho et al., 2021). Among several CTX-M variants, CTX-M-9 type ESBL was dominant at the end of the 1990s, but appeared to be rapidly displaced since 2011 by CTX-M-15 and CTX-M-14 types. (Cantón & Coque, 2006). The *bla*CTX-M-15 has been identified as the most common ESBL gene in environment, livestock, and human-associated *E. coli* (Cantón & Coque, 2006; Zurfluh et al., 2015). The CTX-M-15- and CTX-M-14-type ESBLs (92.5%) were the most predominant ESBLs in the present study, which is similar to the reports from Asia, Africa, Europe, America, Australia, and South Korea (Chen et al., 2014; Iroha et al., 2012; Livermore et al., 2007; Pietsch et al., 2017; Sidjabat et al., 2010; Song et al., 2009).

### Co-harboring of extended-spectrum β-lactamase encoding genes and other antimicrobial resistance genes

It is well known that most of the ESBL-producing pathogenic *E. coli* carry additional antibiotics resistant genes (Park et al., 2022). Here, all ETEC and EPEC strains harboring the *bla*CTX-M gene also carried plasmid-mediated quinolone resistance (PMQR), namely *qnrS1* (98.0%, 48/49) and *qnrS2* (2.0%, 1/49) (Fig. 2). Several previous studies have reported co-existence of PMQR and ESBL genes in *E. coli* (Azargun et al., 2018; Nazik et al., 2011 Viana et al., 2013). The *qnr* genes were highly specific to ETEC strains in this study (Fig. 2). Previous studies have also reported the co-existence of *qnr* and *bla*CTX-M genes in ESBL-producing ETEC, which may be because both genes encoding enterotoxins (LT/ST) and PMQR are plasmid-borne (Gyles et al., 1974; Jiang et al., 2008). However, further research is needed to understand these results better. Likewise, a high prevalence of *qnr* genes among ESBL-producing *E. coli* has been previously reported in South Korea (Park et al., 2007). In addition, most of the EAEC strains harboring the *bla*CTX-M-14 gene also carried the chloramphenicol resistance gene *catA1* (92%, 23/25), folate pathway antagonist gene *sul1*/*dfrA5* (88%, 22/25), and tetracycline gene *tet(A)* (92%, 23/25) (Fig. 2). To confirm the antibiotic resistance attributable to the co-existence of antimicrobial resistance genes, the minimal inhibitory concentrations (MICs) of ciprofloxacin, tetracycline, chloramphenicol, and trimethoprim/sulfamethocxazole were determined (Table 4). The MICs of tetracycline, chloramphenicol, and trimethoprim/sulfamethoxazole were higher than the breakpoints of CLSI and EUCAST guidelines (Table S3) in most of the CTX-M-14 type ESBL-producing EAEC strains carrying *catA1, sul1, dfrA5*, and *tet(A)*. This suggests that the phenotype was consistent with the genotype in these strains.

**Table 4:** Antimicrobial susceptibility of representative ESBL-producing pathogenic *E. coli*

Strains	Patho type	AMR genes							MIC values by E-test (ug/ml)			
		*bla*CTX-M-14	blaCTX-M-15	*qnrS1*	*catA1*	*sul1*	*dfrA5*	*tet(A)*				
									CIP	TET	CHL	SXT
14,188–13	EAEC	O			O	O	O	O	0.012	≥ 256	> 256	> 32
17,528–22		O			O	O	O	O	0.016	≥ 256	> 256	> 32
16,234–19	ETEC		O	O					0.25	2	4	0.64
17,506–31			O	O					0.38	1.5	4	0.47

*catA1,* chloramphenicol; *sul1, dfrA5,* folate pathway antagonist; *tet(A),* tetracycline; *qnrS1,* quinolone; *CIP,* ciprofloxacin; *TET,* tetracycline; *CHL,* chloramphenicol; *SXT,* trimethoprim/sulfamethocxazole

Although *E. coli* is a common commensal gut bacterium, our findings emphasize the high prevalence of ESBL and PMQR gene in pathogenic *E. coli* strains isolated from diarrhea patients of foodborne disease outbreaks. These results suggest that the prevalence of ESBL-producing pathogenic *E. coli* strains may become an important public health concern, highlighting the urgent necessity for monitoring the spread of these foodborne pathogens.

### Supplementary Information

Below is the link to the electronic supplementary material.

Supplementary file1 (DOCX 36 KB)
