# Supplementary material for: Prevalence of CTX-M types among ESBL-producing pathogenic Escherichia coli isolates from foodborne diarrheal patients in Gyeonggi-do, South Korea
Source: Food Sci Biotechnol. 2024 Apr 24;33(12):2825–33. doi: 10.1007/s10068-024-01549-5 (PMC11339195; doi:10.1007/s10068-024-01549-5)
Supplement: Supplementary file 1 — Supplementary file1 (DOCX 36 KB) [file 10068_2024_1549_MOESM1_ESM.docx]

**Supplementary Tables**

**Table S1.** Summary of 80 ESBL producing pathogenic *E. coli* strains isolated in Gyeonggi-do, South Korea from 2014 to 2018

| No.Sample | Time | Strains | Genome Accession | Origin | City | Pathotype |
| --- | --- | --- | --- | --- | --- | --- |
| 1 | July-2014 | 14142-07 | JAWCWY000000000 | Patient-1 | Gwangju | EPEC |
| 2 | Aug-2014 | 14188-02 | JAWCWZ000000000 | Patient-2 | Yangpyeong | EAEC |
| 3 |  | 14188-03 | JAWCXA000000000 | Patient-3 |  | EAEC |
| 4 |  | 14188-07 | JAWCXB000000000 | Patient-4 |  | EAEC |
| 5 |  | 14188-08 | JAWCXC000000000 | Patient-5 |  | EAEC |
| 6 |  | 14188-13 | JAWCXD000000000 | Patient-6 |  | EAEC |
| 7 |  | 14188-14 | JAWCXE000000000 | Patient-7 |  | EAEC |
| 8 |  | 14188-18 | JAWCXF000000000 | Patient-8 |  | EAEC |
| 9 | Sep-2014 | 14261-01 | JAWCXG000000000 | Patient-9 | Hwaseong | EAEC |
| 10 |  | 14261-03 | JAWCXH000000000 | Patient-10 |  | EAEC |
| 11 | Jan-2015 | 15031-01 | JAWCXI000000000 | Patient-11 | Hanam | EPEC |
| 12 |  | 15031-02 | JAWCXJ000000000 | Patient-12 |  | EPEC |
| 13 | Mar-2015 | 15103-22 | JAWCXK000000000 | Patient-13 | Yongin | EPEC |
| 14 | Aug-2016 | 16234-02 | JAWCXL000000000 | Patient-14 | Suwon | ETEC |
| 15 |  | 16234-03 | JAWCXM000000000 | Patient-15 |  | ETEC |
| 16 |  | 16234-07 | JAWCXN000000000 | Patient-16 |  | ETEC |
| 17 |  | 16234-10 | JAWCXO000000000 | Patient-17 |  | ETEC |
| 18 |  | 16234-13 | JAWCXP000000000 | Patient-18 |  | ETEC |
| 19 |  | 16234-15 | JAWCXQ000000000 | Patient-19 |  | ETEC |
| 20 |  | 16234-16 | JAWCXR000000000 | Patient-20 |  | ETEC |
| 21 |  | 16234-18 | JAWCXS000000000 | Patient-21 |  | ETEC |
| 22 |  | 16234-19 | JAWCXT000000000 | Patient-22 |  | ETEC |
| 23 |  | 16234-24 | JAWCXU000000000 | Patient-23 |  | ETEC |
| 24 |  | 16234-26 | JAWCXV000000000 | Patient-24 |  | ETEC |
| 25 |  | 16234-27 | JAWCXW000000000 | Patient-25 |  | ETEC |
| 26 |  | 16234-29 | JAWCXX000000000 | Patient-26 |  | ETEC |
| 27 |  | 16234-32 | JAWCXY000000000 | Patient-27 |  | ETEC |
| 28 |  | 16234-34 | JAWCXZ000000000 | Patient-28 |  | ETEC |
| 29 |  | 16234-35 | JAWCYA000000000 | Patient-29 |  | ETEC |
| 30 |  | 16234-36 | JAWCYB000000000 | Patient-30 |  | ETEC |
| 31 |  | 16234-37 | JAWCYC000000000 | Patient-31 |  | ETEC |
| 32 |  | 16234-39 | JAWCYD000000000 | Patient-32 |  | ETEC |
| 33 |  | 16234-40 | JAWCYE000000000 | Patient-33 |  | ETEC |
| 34 |  | 16234-41 | JAWCYF000000000 | Patient-34 |  | ETEC |
| 35 |  | 16234-43 | JAWCYG000000000 | Patient-35 |  | ETEC |
| 36 |  | 16237-08 | JAWCYH000000000 | Patient-36 |  | ETEC |
| 37 |  | 16237-34 | JAWCYI000000000 | Patient-37 |  | ETEC |
| 38 | Mar-2017 | 17139-02 | JAWCYJ000000000 | Patient-38 | Hwaseong | EPEC |
| 39 | Aug-2017 | 17464-02 | JAWCYK000000000 | Patient-39 | Anseong | EPEC |
| 40 |  | 17464-13 | JAWCYL000000000 | Patient-40 |  | EPEC |
| 41 | Aug-2017 | 17506-01 | JAWCYM000000000 | Patient-41 | Siheung | EAEC |
| 42 |  | 17506-04 | JAWCYN000000000 | Patient-42 |  | ETEC |
| 43 |  | 17506-08 | JAWCYO000000000 | Patient-43 |  | EAEC |

**Table S1. (Continued)**

| No.Sample | Time | Strains | Genome Accession | Origin | City | Pathotype |
| --- | --- | --- | --- | --- | --- | --- |
| 44 | Aug-2017 | 17506-13 | JAWCYP000000000 | Patient-44 | Siheung | EAEC |
| 45 |  | 17506-15-1 | JAWCYQ000000000 | Patient-45 |  | EAEC |
| 46 |  | 17506-15-2 | JAWCYR000000000 | Patient-46 |  | ETEC |
| 47 |  | 17506-18 | JAWCYS000000000 | Patient-47 |  | ETEC |
| 48 |  | 17506-22 | JAWCYT000000000 | Patient-48 |  | EAEC |
| 49 |  | 17506-26 | JAWCYU000000000 | Patient-49 |  | ETEC |
| 50 |  | 17506-29 | JAWCYV000000000 | Patient-50 |  | ETEC |
| 51 |  | 17506-31 | JAWCYW000000000 | Patient-51 |  | ETEC |
| 52 |  | 17506-33 | JAWCYX000000000 | Patient-52 |  | ETEC |
| 53 |  | 17506-37 | JAWCYY000000000 | Patient-53 |  | EAEC |
| 54 |  | 17506-38 | JAWCYZ000000000 | Patient-54 |  | ETEC |
| 55 |  | 17506-41 | JAWCZA000000000 | Patient-55 |  | EAEC |
| 56 |  | 17506-44 | JAWCZB000000000 | Patient-56 |  | ETEC |
| 57 |  | 17506-45 | JAWCZC000000000 | Patient-57 |  | ETEC |
| 58 |  | 17506-47 | JAWCZD000000000 | Patient-58 |  | ETEC |
| 59 |  | 17506-55 | JAWCZE000000000 | Patient-59 |  | ETEC |
| 60 | Aug-2017 | 17528-04 | JAWCZF000000000 | Patient-60 | Gwangju | EAEC |
| 61 |  | 17528-05 | JAWCZG000000000 | Patient-61 |  | ETEC |
| 62 |  | 17528-08-1 | JAWCZH000000000 | Patient-62 |  | EAEC |
| 63 |  | 17528-08-2 | JAWCZI000000000 | Patient-63 |  | ETEC |
| 64 |  | 17528-13 | JAWCZJ000000000 | Patient-64 |  | ETEC |
| 65 |  | 17528-16 | JAWCZK000000000 | Patient-65 |  | ETEC |
| 66 |  | 17528-17 | JAWCZL000000000 | Patient-66 |  | EAEC |
| 67 |  | 17528-18 | JAWCZM000000000 | Patient-67 |  | ETEC |
| 68 |  | 17528-21 | JAWCZN000000000 | Patient-68 |  | EAEC |
| 69 |  | 17528-22 | JAWCZO000000000 | Patient-69 |  | EAEC |
| 70 |  | 17528-23 | JAWCZP000000000 | Patient-70 |  | EAEC |
| 71 |  | 17528-24-1 | JAWCZQ000000000 | Patient-71 |  | EAEC |
| 72 |  | 17528-24-2 | JAWCZR000000000 | Patient-72 |  | ETEC |
| 73 |  | 17528-25 | JAWCZS000000000 | Patient-73 |  | EAEC |
| 74 |  | 17528-39 | JAWCZT000000000 | Patient-74 |  | EAEC |
| 75 |  | 17528-40-1 | JAWCZU000000000 | Patient-75 |  | EAEC |
| 76 |  | 17528-40-2 | JAWCZV000000000 | Patient-76 |  | ETEC |
| 77 | Sep-2017 | 17570-01 | JAWCZW000000000 | Patient-77 | Hwaseong | EPEC |
| 78 | Aug-2018 | 18447-04 | JAWCZX000000000 | Patient-78 | Ansan | ETEC |
| 79 |  | 18447-09 | JAWCZY000000000 | Patient-79 |  | EPEC |
| 80 |  | 18447-15 | JAWCZZ000000000 | Patient-80 |  | EPEC |

**Table S2. Antimicrobial susceptibility of ESBL-producing *E. coli* isolates using VITEK 2 system**

| Antimicrobial class | Antimicrobial agent | No. of ESBL-producing pathogenic *E.coli* isolates | | | | | | | | |
| --- | --- | --- | --- | --- | --- | --- | --- | --- | --- | --- |
|  |  | EAEC (n=26) | | | EPEC (n=10) | | | ETEC (n=44) | | |
|  |  | R | I | S | R | I | S | R | I | S |
| Penicillins | Ampicillin | 26 | 0 | 0 | 10 | 0 | 0 | 44 | 0 | 0 |
| Penicillins+ß-lactamase inhibitors | Amoxicillin/Clavulanic Acid | 0 | 0 | 26 | 1 | 2 | 7 | 1 | 2 | 41 |
|  | Ampicillin/Sulbactam | 17 | 0 | 9 | 5 | 1 | 4 | 10 | 0 | 34 |
| Non-extended spectrum cephalosporins (1st generation cephalosporins) | Cefalotin | 26 | 0 | 0 | 10 | 0 | 0 | 44 | 0 | 0 |
|  | Cefazolin | 26 | 0 | 0 | 10 | 0 | 0 | 44 | 0 | 0 |
| Cephamycins | Cefotetan | 0 | 0 | 26 | 0 | 1 | 9 | 0 | 0 | 44 |
|  | Cefoxitin | 0 | 0 | 26 | 1 | 0 | 9 | 2 | 0 | 42 |
| Extended spectrum cephalosporins (3rd generation cephalosporins) | Cefotaxime | 25 | 1 | 0 | 9 | 1 | 0 | 43 | 1 | 0 |
|  | Ceftriaxone | 13 | 13 | 0 | 8 | 2 | 0 | 37 | 7 | 0 |
| Cabapenems | Imipenem | 0 | 0 | 26 | 0 | 0 | 10 | 0 | 0 | 44 |
| Aminoglycosides | Amikacin | 0 | 0 | 26 | 0 | 0 | 10 | 0 | 1 | 43 |
|  | Gentamicin | 1 | 0 | 25 | 0 | 0 | 10 | 3 | 0 | 41 |
| Quinolones | Nalidixic Acid | 2 | 0 | 24 | 2 | 0 | 8 | 5 | 0 | 39 |
| Fluoroquinolones | Ciprofloxacin | 0 | 8 | 18 | 0 | 3 | 7 | 4 | 15 | 25 |
| Tetracyclines | Tetracycline | 15 | 0 | 11 | 5 | 0 | 5 | 11 | 0 | 33 |
| Phenicols | Chloramphenicol | 16 | 1 | 9 | 3 | 5 | 2 | 8 | 25 | 11 |
| Folate Pathway inhibitors | Trimethoprim/Sulfamethocxazole | 16 | 0 | 10 | 7 | 0 | 2 | 10 | 0 | 34 |

The interpretation of resistant (R), intermediate (I) and sensitive (S) was based on the criteria issued by the Clinical and Laboratory Standards Institute.

**Table S3.** List of antimicrobial agents, their subclasses, MIC range of tested concentration, and breakpoints for susceptibility testing

| Antimicrobial subclass | | Antimicrobial agent  (Abbreviation) | MIC range of tested concentration  (㎍/mL) | | ^*^CLSI | EUCAST |
| --- | --- | --- | --- | --- | --- | --- |
|  |  |  |  |  | Breakpoints (㎍/mL) | |
| Fluoroquinolones | Ciprofloxacin (CIP) | | | 0.002-32 | ≥4 | >0.5 |
| Tetracyclines | Tetracycline  (TET) | | | 0.016-256 | ≥16 | >0.5 |
| Phenicols | Chloramphenicol  (CHL) | | | 0.016-256 | ≥32 | >8 |
| Folate Pathway  inhibitors | Trimethoprim/Sulfamethocxazole  (SXT) | | | 0.002-32 | ≥4/72 | >4 |

^*^CLSI, Clinical and Laboratory Standards Institute guidelines, EUCAST, European Committee on Antimicrobial Susceptibility Testing guidelines
